# Fish consumption and its possible preventive role on the development and prevalence of metabolic syndrome - a systematic review

**DOI:** 10.1186/1758-5996-6-112

**Published:** 2014-10-17

**Authors:** Christine Tørris, Marianne Molin, Milada Cvancarova Småstuen

**Affiliations:** Oslo and Akershus University College, Oslo, Norway; Institute of Basic Medical Sciences, University of Oslo, Blindern, Oslo, Norway

**Keywords:** Metabolic syndrome, Insulin resistance, Diet, Fish intake, Seafood, Consumption of fish, Systematic review

## Abstract

Metabolic syndrome (MetS) has a huge impact on public health, and today lifestyle interventions remain the primary mode for MetS therapy. It is therefore important to elucidate the possible preventive effects of diet and foods, and their MetS-related health implications. To examine how fish consumption affects the development and prevalence of MetS, we systematically reviewed cross-sectional, prospective cohort, and intervention studies conducted among adults (humans) and, reporting consumption of fish or seafood as being related to MetS (prevalence or incidence), where MetS was defined via an established definition. The literature search in PubMed identified 502 citations, and after screening, 49 full-text articles were retrieved and assessed for eligibility. After excluding duplicates and those not meeting the inclusion criteria, seven studies from Croatia, Finland, France, Iceland, Iran, Korea, and US were included. Four studies (one follow-up and three cross-sectional) found associations between fish consumption and MetS (three among men, and one among women), suggesting that fish consumption may prevent or improve metabolic health and have a protective role in MetS prevention. This protective role might be related to gender, and men may benefit more from the consumption of fish. However, lack of controlling for potential confounders may also inflict the results. Additional research is required to further explore fish consumption and its potential role in improving or reversing MetS and its components.

## Introduction

Metabolic syndrome (MetS) is a cluster of risk factors for cardiovascular disease (CVD) and diabetes mellitus type 2 (DM2) with metabolic abnormalities including abdominal obesity, dyslipidaemia, hyperglycaemia, and hypertension [1, 2]. Several definitions and diagnostic criteria for MetS have been proposed, the latest by the new Joint Interim Societies (JIS) [2]. MetS has been associated with a doubling of CVD risk as well as a 5-fold increased risk of DM2 [3]. The syndrome affects public health, and the increased risk of morbidity and mortality is profound [4].

Consumption of fish has been identified as a protective factor against several types of disease. Over the past decades a considerable amount of literature has been published on CVD and the benefits of fish consumption [5, 6]. Today, lifestyle interventions remain the primary therapy for MetS, and it is important to emphasize the role of diet and food, such as fish, and their possible MetS-related health implications. So far, many studies have focused on single components in marine nutrients, such as n-3 fatty acids [7, 8]. However, few studies have investigated associations between fish consumption and MetS development and prevalence. This study primarily aims to examine how the consumption of fish affects the development and prevalence of MetS. This research question was explored by reviewing cross-sectional, prospective cohort, and intervention studies conducted among adults (humans) and reporting consumption of fish or seafood as being related to MetS (prevalence or incidence), where MetS was defined via an established definition.

## Methods

Literature search was performed in PubMed to identify published studies examining associations between consumption of fish among humans as the exposure, versus the development and prevalence of MetS as the outcome. Combined search terms were *fish, seafood, intake,* and *consumption* as exposure search terms, and *metabolic syndrome* or *insulin resistance* as outcome search terms. The last search was performed June 1^st^ 2014.

**Figure 1:**
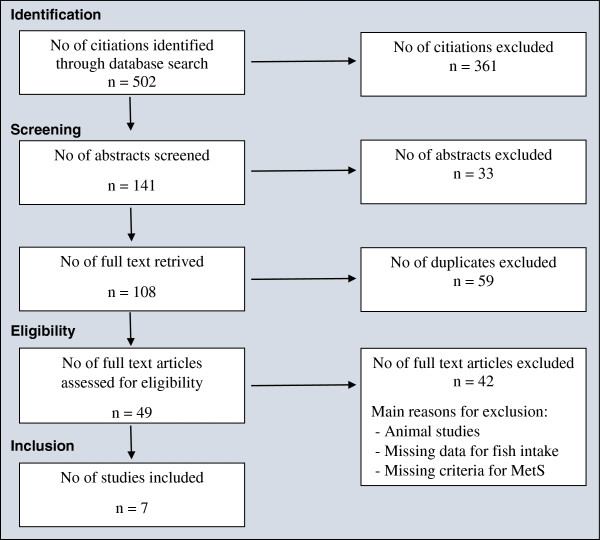
**Flow of studies through the different phases of this systematic review.**

Potential abstracts and full-text articles were screened before removing duplicates. Full-text articles were assessed for eligibility, and seven studies were included in this review after exclusion. The selection process is illustrated via a flow diagram (Figure 1).

All cross-sectional, prospective cohort, and intervention studies considered for inclusion were conducted among adults (humans), and reported consumption of fish or seafood related to MetS (prevalence or incidence), where MetS was defined via an established definition. Prospective cohort studies were considered eligible for inclusion if they had at least one year of follow-up and involved general populations. Insulin resistance syndrome (IRS) was accepted as an outcome factor and considered for inclusion when using the same definition as in MetS. Abstracts, letters, or reviews were not included, but were inspected for additional references that meet the inclusion criteria. Reference lists of the included studies and relevant published reviews were examined to identify additional papers for possible inclusion.

The search was restricted to papers written in English, and animal studies were excluded. Full-text of the article was retrieved whenever there was uncertainty about the study’s match with the inclusion criteria. The included studies were assessed according to the quality of the study design and methods, measurements of MetS and fish consumption, and the statistical analysis. The review procedure was carried out in accordance with the PRISMA statement for review reporting [9], and a protocol of the study selection was made.

The data collected from the studies included: reference, country where the study was performed, design, aim, participants’ age and sex, (baseline age and duration of follow-up for prospective studies), sample size, methods of measurement, variables adjusted for in the analysis, multivariate adjusted OR with a 95% CI for the lowest versus the highest consumption of fish, and MetS incidence or prevalence.

## Results

The literature search identified 502 citations, and after screening titles and abstracts, 49 full-text articles were retrieved and assessed for eligibility. Finally, seven studies investigating associations between fish consumption and MetS were included in this review. The criteria for diagnosing MetS for the included studies are shown in Table 1, and study characteristics are provided in Table 2. The included studies comprise of one intervention study from Iceland [10], one follow-up study from Korea [11], and five cross-sectional studies [12–16]- three conducted in Europe (Finland [12], Croatia [14], France [15]), one in USA [13], and one in Iran [16].

**Table 1:** **Different criteria for diagnosing metabolic syndrome (MetS) used in studies included in this review**

	Criteria	Study	Men	Women
**Waist**	IDF [22]	Pasalic et al., 2011 [14]	≥ 94 cm	≥ 80 cm
	IDF [17]	Ramel et al., 2009 [10]		
	JIS [2]	Zaribaf et al., 2014 [16]		
	ATP III [20]	Kouki et al., 2011 [12]	> 102 cm	> 88 cm
	ATP III [21]	Lai et al., 2013 [13]		
	ATP III [20]	Ruidavets et al., 2007 [15]		
	Alternative [19]	Baik et al., 2010 [11]	≥ 85 cm	≥ 80 cm
**S-HDL cholesterol**	ATP III [18]	Baik et al., 2010 [11]	< 1.0 mmol/L (40 mg/dL)	< 1.3 mmol/L (50 mg/dL)
	ATP III [20]	Kouki et al., 2011 [12]		
	ATP III [21]	Lai et al., 2013 [13]		
	IDF [22]	Pasalic et al., 2011 [14]		
	IDF [17]	Ramel et al., 2009 [10]		
	ATP III [20]	Ruidavets et al., 2007 [15]		
	JIS [2]	Zaribaf et al., 2014 [16]		
**S-triglyceride**	ATP III [18]	Baik et al., 2010 [11]		
	ATP III [20]	Kouki et al., 2011 [12]		
	ATP III [21]	Lai et al., 2013 [13]		
	IDF [22]	Pasalic et al., 2011 [14]	> 1.7 mmol/L (150 mg/dL)	
	IDF [17]	Ramel et al., 2009 [10]		
	ATP III [20]	Ruidavets et al., 2007 [15]		
	JIS [2]	Zaribaf et al., 2014 [16]		
**Blood pressure**	ATP III [18]	Baik et al., 2010 [11]		
	ATP III [20]	Kouki et al., 2011 [12]		
	ATP III [21]	Lai et al., 2013 [13]		
	IDF [22]	Pasalic et al., 2011 [14]	SBP ≥130 mm Hg	
	IDF [17]	Ramel et al., 2009 [10]	DBP ≥85 mm Hg	
	ATP III [20]	Ruidavets et al., 2007 [15]		
	JIS [2]	Zaribaf et al., 2014 [16]		
**Fasting S-glucose**	ATP III [18]	Baik et al., 2010 [11]	≥ 5.6 mmol/ L (100 mg/dL)	
	IDF [22]	Pasalic et al., 2011 [14]		
	IDF [17]	Ramel et al., 2009 [10]		
	JIS [2]	Zaribaf et al., 2014 [16]		
	ATP III [20]	Kouki et al., 2011 [12]	≥ 6.1 mmol/l (110 mg/dL)	
	ATP III [21]	Lai et al., 2013 [13]		
	ATP III [20]	Ruidavets et al., 2007 [15]		

ATP III: Adult Treatment Panel III; the presence of three criteria must be fulfilled to diagnose MetS. IDF: International Diabetes Foundation; the criteria of waist (population specific) and two of the other criteria must be fulfilled to diagnose MetS.

JIS: Joint Interim Statement; the criteria of waist (population specific) and two of the other criteria must be fulfilled to diagnose MetS.SBP: Systolic blood pressure. DBP: Diastolic blood pressure.

**Table 2:** **Characteristics of studies on fish consumption and metabolic syndrome (MetS), organized by study design (intervention, prospective, cross-sectional)**

Reference	Country/design	Aim	Population	Diet	Adjustments	Results adjusted OR (95% CI) incidence/prevalence of MetS
Ramel et al., 2009 [10]	Iceland RCT, part of SEAFOODplus YOUNG study 8w parallel intervention	Investigate whether cod consumption increases weight loss and CVD risk factors	n = 126, 20–40 y, overweight healthy, no gender distribution given	Energy-restricted diets (−30%), identical macronutrient composition, different amounts of cod (control group: no seafood, group 1: 150 g cod 3x/w, group 2: 150 g cod 5x/w)	In linear model with waist (baseline anthropometric measure, gender, diet group), and with blood variables (baseline anthropometric measure, weight loss, gender, diet group)	No association
Baik et al., 2010 [11]	Korea 3 y follow up Korean Genome Epidemiology Study	Investigate effect of fish and n-3 fatty acid intake on incidence of MetS	n = 3504, 40–69 y 52% men, free of MetS and CVD at baseline	FFQ Average fish intake was grouped (<1/week, 1-4/week, 5-6/week and daily)	Age, BMI, income, occupation, marital status, education, smoking, alcohol, physical activity, dietary intake (energy, fat, fibre, red meat, dairy products, sweetened carbonated beverage, multivitamin), health (diabetes, hypertension, menopausal status, postmenopausal hormone use)	Men: Average daily intake of fish (40–70 g) reduced the risk of having MetS, compared with eating fish less than once a week OR 0.43 (0.23-0.83) Women: No associations
Kouki et al., 2011 [12]	Finland Cross-sectional, DR’s EXTRAStudy	Studie associations between food items and nutrients versus the risk of having MetS	n = 1 334, 57–78 y 50% men Representative population sample	4-day food record Consumption of fish divided into tertiles (<18.5, 18.5–59.5, (>59.5 g/day)	a, Age, alcohol consumption, smoking b, Further for education, VO_2_max	Men: the middle and highest tertile had almost half the risk of having MetS, compared to lowest tertile. a, t2: 0.51 (0.33–0.77) t3: 0.53 (0.35–0.81)b, t2: 0.52 (0.32–0.83) t3: 0.63 (0.40–1.00)Women: No association
Lai et al., 2013 [13]	Cross-sectional National Heart, Lung, and Blood Institute Family Heart Study	Assess association between dietary omega-3 fatty acids and MetS prevalence	n = 4941, mean age (SD) 52.1 (13.9) y 46% men	FFQ (fish consumption classified as 0, 1, 2, ≥3 servings/ week)	Age, gender, race, alcohol intake, smoking, exercise, TV watching, energy intake, multivitamin use, fruits/vegetables intake, fibre intake, risk group	No association
Pasalic et al. 2011 [14]	Croatia Cross-sectional	Investigate prevalence of MetS, and influence of lifestyle habits between continental and Mediterranean–Adriatic centres	n = 320, 70–90 y, 44% men	FFQ Consumption of fish grouped into never/rarely or monthly	Centres	No association
Ruidavets et al., 2007 [15]	France Cross-sectional	Analyse relation between food groups and frequency of IRS	n = 912, 100% men 45–64 years randomly selected	3-day food record fish consumption divided into tertiles	Age, centre, physical activity, education, smoking habits, alcohol consumption, drugs (hypertension, dyslipidaemia), energy intake (without alcohol), dieting, and diet quality index	A negative association between fish intake and prevalence of MetS OR 0.57 (0.38-0.86), when comparing highest tertile to lowest
Zaribaf et al., 2014 [16]	Iran Cross-sectional	Association between fish consumption and metabolic syndrome and its components	n = 420, 100% women, >30 y	FFQ fish consumption divided into tertiles	Age, energy intake, physical activity, socioeconomic status, medication use, marital, menopausal status, dietary intake (red meat, grains, fruits, vegetables, legume, nuts, dairy products, fibre, oils), BMI	Individuals in the highest tertile of fish intake were less likely to have MetS, compared to those in the lowest tertile OR 0.04 (0.004–0.61)

BMI: Body Mass Index; CI: confidence interval; CVD: cardiovascular disease; FFQ: Food Frequency Questionnaire; DBP: diastolic blood pressure; IRS: insulin resistance syndrome; MetS: metabolic syndrome; OR: odds ratio; RCT: randomized clinical trial; SBP: systolic blood pressure; WC: waist circumference.

### Intervention studies

The intervention study was carried out in Iceland [10], as part of the Icelandic arm of the SEAFOODplus YOUNG study. The aim was to investigate whether cod consumption increased weight loss and improved cardiovascular (CV) risk factors during an eight-week energy-restricted diet (−30%). Overweight/obese, but otherwise healthy young adults aged 20–40 years were recruited through advertisements (n = 126). One hundred (79%) participants completed the eight-week intervention with an identical macronutrient composition and various amounts of cod (control: no seafood; group 1: 150 g cod three times a week; group 2: 150 g cod five times a week). MetS was defined according to the definition from the International Diabetes Federation (IDF) [17] (Table 1), and compliance to seafood consumption was assessed with a validated food frequency questionnaire (FFQ). Different variables were included in linear models when analyzing anthropometric measures and blood variables, waist (baseline anthropometric measure, gender, diet group), and blood variables (baseline anthropometric measure, weight loss, gender, diet group). After eight-weeks of intervention, MetS prevalence dropped from 29% to 21% in the total study population, in addition to a reduction in the components of MetS: waist circumference (WC) (5.0 ± 3.2 cm, P < 0.001), systolic (3.4 ± 8.9 mmHg, P = 0.001) and diastolic blood pressure (BP) (2.4 ± 6.9 mmHg, P < 0.001), and triglycerides (TG) (1.26 ± 0.567 mmol/L, P = 0.03). The decrease in WC was greater (−3.4 cm, P < 0.001) among subjects consuming cod (150 g) 5x/week, compared to the control group. However, the BP was slightly higher in the groups consuming fish, compared to the control group, with systolic BP 5.3 mmHg higher (P = 0.005) for those consuming cod 5x/week and diastolic BP 3.2 mmHg higher (P = 0.022) for those consuming cod 3x/week. Blood lipids and glucose were not affected in the cod consumption groups [10].

### Prospective studies

The follow-up study [11] included male and female Koreans (n = 3504) aged 40–69 years from the Korean Genome Epidemiology Study, a population-based prospective cohort study. Participants with MetS at baseline were excluded. MetS was defined according to the definition from the Adult Treatment Panel III (ATP III) [18], except for WC, where alternative criteria were used for the appropriate waist cut-off points [19] (Table 1). Associations between average frequency of fish consumption (sum of dark- or white-meat fish and canned tuna), and incidence of MetS were investigated. The average frequency of fish intake was grouped into <1/week, 1-4/week, 5-6/week, and daily, with the lowest intake category (<1/week) used as reference. Potential confounding factors were adjusted for (Table 2). The study reported a 17% incidence of MetS over the follow-up period, whereof 57% were men. The results showed that the adjusted risk of having MetS decreased to less than half (OR 0.43, 95% CI 0.23-0.83) among men who consumed fish daily, compared with those who consumed fish less than once a week. Furthermore, fish consumption was significantly associated with a lower TG level (P < 0.01) and an elevated high-density lipoprotein (HDL) cholesterol level (P < 0.01) among men. However, no significant association was found among women [11].

### Cross-sectional studies

In a large Finnish population (n = 1334) associations between food and nutrients and the risk of having MetS were studied [12], where MetS was defined according to the ATP III definition [20] (Table 1). Baseline data from a population-based randomized controlled trial (RCT), the Dose Responses to Exercise Training (DR’s EXTRA) study, was used. Dietary intake was assessed by a four-day food record, which included three weekdays and one weekend day, and utilized a picture booklet of portion sizes. MetS prevalence in the Finnish population was 28% among men and 25% among women. Individuals with MetS had a lower consumption of fish (g/d) compared to those without MetS, although the difference was only significant among men (P = 0.001). After adjusting for age, smoking status, and alcohol consumption, there was an inverse association between fish consumption (10 g/d) and MetS in men (OR 0.97, 95% CI 0.94–1.00). After further adjusting for cardiorespiratory fitness (VO_2max_), the association was no longer significant. When dividing fish consumption into tertiles, men in the middle (18.5–59.5 g/day) and highest tertile (>59.5 g/day) had almost half the risk of having MetS, compared to those in the lowest tertile (<18.5 g/day) (P = 0.002), when adjusting for age, smoking status, and alcohol consumption. This association remained significant after additional adjustments for VO_2_max and education (0.04). No significant associations was found in women [12].

In a US population, the associations between fish consumption and MetS were studied cross-sectionally in a large population (N = 4941) from the National Heart, Lung, and Blood Institute (NHLBI) Family Heart Study [13]. MetS was defined according to the ATP III definition [21] (Table 1), and frequency of fish intake was obtained from a FFQ and classified as 0, 1, 2, or 3 or more servings per week. Potential confounding factors were adjusted for (Table 2). In this population, with a MetS prevalence of 21.0%, no association was found between fish consumption and MetS prevalence. A higher consumption of fish was, however, related to a higher TG, compared to no fish consumption (155.8 mg/dL vs. (144.6 mg/dL) [13].

In a healthy elderly Croatian population (aged 70–90 years) from four centres (two continental and two coastal), MetS prevalence and characteristics were investigated [14]. MetS was defined according to the IDF definition [22] (Table 1). Dietary intake was assessed by food questionnaires related to eating habits. Consumption of fish was grouped monthly (or more frequent) and included pelagic fish (anchovy and/or mackerel and/or tuna) and freshwater fish (carp and/or catfish and/or trout). In this elderly population, with a 60-70% prevalence of MetS, there was no significant difference in MetS prevalence and fish consumption even after adjusting for centre-related variables. Nevertheless, a higher prevalence of men with high HDL-cholesterol was observed among those frequently consuming pelagic fish, compared to those without frequent fish consumption (P = 0.03) [14]. MetS prevalence was significantly lower (P = 0.05) among participants from small centres (59%), who consumed more lard, freshwater fish, spirits, and wine, versus those from large centres (70%), although only wine consumption reached significance (P = 0.05). Still, there was no difference between continental and coastal centres or in prevalence of MetS among men and women [14].

In a French population, consisting only of men (aged 45–64 years) randomly selected from three regions in France, the relation between food groups and frequency of IRS were examined [15]. IRS was defined according to the ATP III definition [20] (Table 1), and dietary intake was assessed via a three-day food record. The consumption of fish (g/d) (sea fish, river fish, and seafood) was grouped into tertiles, due to the high percentage of non-fish eaters, and potential confounding factors were adjusted for (Table 2). In this population IRS prevalence was 24%, however, the prevalence reached 29% when fish consumption was below the median. A negative association between IRS and fish consumption was found, and proportions of IRS decreased along tertiles for fish consumption (adjusted OR 0.57, 95% CI 0.38-0.86) in the highest tertile of fish consumption [15].

Finally, the cross-sectional study from Iran [16] aimed to identify associations between fish consumption and MetS and its components in a population of women. MetS was defined according to the JIS definition [2] (Table 1). Female nurses (aged >30 years) were randomly selected from various hospitals in Iran (n = 420). Fish consumption was assessed using a FFQ and calculated by the summation of fish and tuna consumption, based on tertile cut-off points of energy-adjusted fish intake. The analysis adjusted for potential confounding factors (Table 2), and uncovered an 8% prevalence of MetS among this population of young women. The research revealed that high fish consumption was inversely associated with MetS, and some of its features (low HDL-cholesterol, hypertriglyceridemia, high BP). Women in the highest tertile of fish consumption were less likely to have MetS, compared with their counterparts in the lowest tertile (OR 0.35, 95% CI 0.14-0.88), and the association was strengthened when adjusting for socio-demographic and dietary variables, and BMI (OR 0.04, 95% CI 0.004-0.61) [16].

## Discussion

In this systematic review, we explore how fish consumption affects MetS development and prevalence. A total of seven studies were found to be eligible for inclusion in the review, of which four studies revealed associations between fish consumption and MetS: one follow-up study [11] and three cross-sectional studies [12, 15, 16]. However, only one study found associations among women [16]. Despite data scarcity, the results suggest that fish consumption may have a preventive role in MetS development and possibly improve metabolic health, although this protective role might be gender-related.

### Prevalence and incidence of metabolic syndrome

In five of the studies included in this review [10–13, 15], there were small differences in MetS prevalence (21%-29%). The prevalence in the French study, reached 29% when fish consumption was below median consumption [15]. In the group from Iceland, MetS prevalence was 29% [10], which is surprisingly low for an overweight/obese population, although they were young and regarded as otherwise healthy [10]. The review did, however, include two studies that differed in MetS prevalence: an 8% prevalence of MetS was reported in the young population of women from Iran [16], while a 60-70% MetS prevalence was reported for the elderly population from Croatia [14]. Interestingly, the prevalence in the latter study was significantly lower (P = 0.05) among participants from small centres (59%), where they consumed more lard, freshwater fish, spirits, and wine in comparison with those from large centres (70%). However, a significant difference was only seen among participants in the wine consuming groups, with a lower prevalence of MetS in the group reporting daily wine consumption (P = 0.05). Still, there was no difference between continental and coastal centres or in prevalence among men and women [14]. Some studies, not included in this review, found an increased prevalence of MetS among men [12, 23–25], some among women [26, 27], and some studies found no gender differences [14, 28, 29].

The Korean follow-up study [11] included in this review reported a 17% incidence of MetS over the follow-up period, whereof 57% were men. An inverse association between daily fish consumption and MetS incidence was found among men, but not among women.

### Metabolic syndrome, fish consumption and gender

Three of the five cross-sectional studies included in this review uncovered associations between fish consumption and MetS [12, 15, 16]. Nevertheless, two studies [12, 15] found associations only among men, while one study found associations among women [16]. In the Finnish population both men and women with MetS had a lower fish consumption than those without MetS [12]. Furthermore, men in the highest tertile of fish consumption had almost half the risk of having MetS, compared to those in the lowest third (P = 0.002) [12]. In the French study [15], where the population consisted only of men, the risk of developing IRS was twice as high for those whose fish consumption fell below the median value (33 g/day), versus those who consumed above the median value (OR 0.51, 95% CI 0.36-0.71) [15]. In the study from Iran [16], which only included women, high fish consumption was inversely associated with MetS, but here the fish consumption was based on tertile cut-off points of energy-adjusted fish consumption [16]. Only two cross-sectional studies failed to discern an association between fish consumption and MetS prevalence [13, 14], although the Croatian study found a higher prevalence of men with high HDL-cholesterol among those frequently consuming pelagic fish compared to those without frequent fish consumption [14]. However, the study [14] did not differ between high and low consumers of fish, and it is therefore not possible to explore whether the results were associated with high or low fish consumption.

Differences in results found among men and women have been previously observed in studies investigating MetS and dietary patterns containing fish. A study from Spain (n = 808) [30] found an inverse association between Mediterranean diet (Med Diet) and risk of having MetS, although the association was significant only in men (P = 0.005), not in women (P = 0.056). This perhaps suggests that fish consumption may prevent or improve metabolic health and decrease MetS prevalence, and that differences in associations may be related to gender and lifestyle factors. A Korean study investigating associations between MetS and different dietary patterns in women found an inverse association between MetS and a healthy dietary pattern containing fish (OR 0.58, 95% CI 0.50-0.91) for highest versus lowest quartile. When stratifying by menopausal status, the inverse association was only significant among postmenopausal women (OR 0.60, 95% CI 0.40-0.86) for highest versus lowest quartile [31]. This may suggest gender differences, at least until menopause, and that premenopausal woman may have some natural protection against MetS, possibly from higher oestrogen levels and the ability of oestrogen to decrease inflammation and reduce the glucocorticoid response [32].

In women, parity and having a higher number of children have been associated with higher rates of MetS [33], and an increased prevalence with age has also been observed [31]. None of the included studies adjusted for parity or length of lactation in women, possibly resulting in different result among women. Lactation imposes a metabolic burden on the mother due to an increased energy requirement [34, 35], and changes that occur during pregnancy (visceral fat accumulates, insulin resistance, increased lipid and TG levels) may reverse more quickly and more completely with lactation [36–38]. Moreover, prolonged lactation may be associated with a healthier metabolic profile and body composition, especially lipid levels and waist-to-hip ratios [38].

### Metabolic syndrome and lean fish

In the intervention study from Iceland [10], the effect of lean fish (cod) was examined in an overweight/obese population. The intervention resulted in a drop in MetS prevalence among the total study population, although blood lipids and glucose were not affected by consumption of cod. This reduction in MetS prevalence may therefore be attributed to weight loss rather than the consumption of cod. However, the decrease in WC was greater among the cod consuming groups, compared to the control group [10].

A Spanish intervention study investigating the effect of white fish on CV risk factors in patients with MetS found that lean fish (seven servings of hake per week) reduced both WC and diastolic BP [39]. One possible explanation for the protective effects is that lean fish, such as cod, are considered a superior source of proteins. Proteins in fish have been associated with body weight reduction, through their positive effect on satiety, compared to other animal proteins [40]. The proteins in fish are easily digestible and rich in essential amino acids. Dietary proteins regulate lipid metabolism, depending on the quantity of proteins and composition of the diet, and have been seen to slow absorption and synthesis of lipids, and promote the lipid excretion [41]. Animal studies have suggested that fish protein may have multiple effects on plasma and liver lipids [42], and that consumption of fish protein might have beneficial effects on hyperglycaemia and hyperlipidaemia [43, 44]. An improved insulin sensitivity has also been seen in insulin-resistant men and women consuming proteins from cod, compared to other animal proteins [45].

### Metabolic syndrome, lifestyle and dietary pattern

Lifestyle habits, like dietary and behaviour lifestyle, as well as socioeconomic status (SES) appear to influence the prevalence and development of MetS across gender, age, and race/ethnicity [3]. Over the years, a number of individual foods and nutrients (e.g., fats, meat, fruits, vegetables, fish, dietary fibre) have been associated with MetS, although no single nutrient has been proposed entirely responsible. MetS has been associated with an unhealthy lifestyle, and positive associations with a diet high in processed foods such as refined grains, rice, potato chips, and pancakes have been observed [46]. When different healthy foods are consumed together, there may be an additive effect, and in one of the cross-sectional studies included in this review [14], an inverse association with MetS was found when fish was consumed with legumes, nuts and berries monthly or more frequently. However, this was seen only among men [14]. Such possible additive effects have also been observed when fish is consumed together with dairy products and grain [15], or when associations between MetS and dietary patterns containing fish have been investigated in follow-up studies such as Healthy Diet [47] and Med Diet [48, 49]. Still, not all follow-up studies point to associations between MetS and dietary patterns containing fish [50]. Of the cross-sectional studies investigating associations between MetS and dietary patterns containing fish, beneficial associations were seen in six studies [25, 27, 30, 31, 51, 52], while ten cross-sectional studies failed to establish such association [23, 24, 26, 28, 29, 46, 53–56].

When examining dietary patterns, the pattern can be either a known dietary pattern used earlier in other studies or a dietary pattern revealed in the actual population. Dietary pattern analysis may capture the effects of dietary exposure, which often are lost in single nutrient or food analyses. However, the use of dietary patterns may be misleading and may hide positive effects of different foods when the dietary intake is summarized into categories or dietary patterns. Different food or food groups may also work against each other, such as when healthy food is placed in a group with unhealthy food, for example when fish and shellfish and the consumption of meat, processed meat, mayonnaise, and eggs are placed into one category [23]. Still, the effects of higher fish consumption may be associated with a healthier lifestyle. Thus, other factors included in a healthy lifestyle such as other healthy foods and a higher level of physical activity may be confounding factors. Of the studies included in this review, most contained some form of adjustment for diet and physical activity.

In the US population included in this review [13], a higher TG level was observed in those consuming fish versus those with no fish consumption. However, the study’s method did not account for how the food is cooked, the positive health effects may diminish or vanish depending on how the meal is prepared, as has been demonstrated in fried fish in studies concerning CVD [57] and DM2 [58]. Therefore, any association may be linked to foods such as fish, to the dietary pattern, or to additive effects of different foods consumed. Today, lifestyle interventions remain the primary therapy for MetS [59], and it is therefore important to focus on lifestyle factors such as diet and different foods such as fish and their possible MetS-related health implications.

### The use of different definitions of metabolic syndrome

Most of the studies included in this review are European, but inter-comparability is complicated by the various MetS criteria employed in the studies (Table 1). Moreover, different definitions may lead to higher or lower MetS prevalence, and higher prevalence of MetS has been seen in studies using the JIS definition [60]. Different populations may also have differences in the amount of abdominal fat, and it is therefore recommended to use a population- and country-specific definition for elevated WC as measures for abdominal obesity [2]. In the included Korean study [11], the European criteria for WC was considered too large. Alternative criteria based on national survey data in Korea was therefore used as the appropriate waist cut-off point for central obesity [11].

## Conclusions

The results from this systematic review suggest that fish consumption may have a protective role in MetS prevention, and suggest that fish consumption may prevent or improve metabolic health. With the rapidly rising prevalence of MetS, further investigation is warranted to establish the ability of fish to improve or reverse MetS and its components, especially regarding any associations according to gender and lean fish.

## References

[1] Alberti KG, Zimmet P, Shaw J (2005). The metabolic syndrome–a new worldwide definition. Lancet.

[2] Alberti KG, Eckel RH, Grundy SM, Zimmet PZ, Cleeman JI, Donato KA, Fruchart JC, James WP, Loria CM, Smith SC (2009). Harmonizing the metabolic syndrome: a joint interim statement of the international diabetes federation task force on epidemiology and prevention; national heart, lung, and blood institute; american heart association; world heart federation; international atherosclerosis society; and international association for the study of obesity. Circulation.

[3] Cornier MA, Dabelea D, Hernandez TL, Lindstrom RC, Steig AJ, Stob NR, Van Pelt RE, Wang H, Eckel RH (2008). The metabolic syndrome. Endocr Rev.

[4] Potenza MV, Mechanick JI (2009). The metabolic syndrome: definition, global impact, and pathophysiology. Nutr Clin Pract.

[5] Raatz SK, Silverstein JT, Jahns L, Picklo MJ (2013). Issues of fish consumption for cardiovascular disease risk reduction. Nutrients.

[6] Panagiotakos DB, Zeimbekis A, Boutziouka V, Economou M, Kourlaba G, Toutouzas P, Polychronopoulos E (2007). Long-term fish intake is associated with better lipid profile, arterial blood pressure, and blood glucose levels in elderly people from Mediterranean islands (MEDIS epidemiological study). Med Sci Monit.

[7] Robinson LE, Mazurak VC (2013). N-3 polyunsaturated fatty acids: relationship to inflammation in healthy adults and adults exhibiting features of metabolic syndrome. Lipids.

[8] Robinson LE, Buchholz AC, Mazurak VC (2007). Inflammation, obesity, and fatty acid metabolism: influence of n-3 polyunsaturated fatty acids on factors contributing to metabolic syndrome. Appl Physiol Nutr Metab.

[9] Moher D, Liberati A, Tetzlaff J, Altman DG (2009). Preferred reporting items for systematic reviews and meta-analyses: the PRISMA statement. BMJ.

[10] Ramel A, Jonsdottir MT, Thorsdottir I (2009). Consumption of cod and weight loss in young overweight and obese adults on an energy reduced diet for 8-weeks. Nutr Metab Cardiovasc Dis.

[11] Baik I, Abbott RD, Curb JD, Shin C (2010). Intake of fish and n-3 fatty acids and future risk of metabolic syndrome. J Am Diet Assoc.

[12] Kouki R, Schwab U, Hassinen M, Komulainen P, Heikkila H, Lakka TA, Rauramaa R (2011). Food consumption, nutrient intake and the risk of having metabolic syndrome: the DR’s EXTRA Study. Eur J Clin Nutr.

[13] Lai YH, Petrone AB, Pankow JS, Arnett DK, North KE, Ellison RC, Hunt SC, Djousse L (2013). Association of dietary omega-3 fatty acids with prevalence of metabolic syndrome: the national heart, lung, and blood institute family heart study. Nutr Clin.

[14] Pasalic D, Dodig S, Corovic N, Pizent A, Jurasovic J, Pavlovic M (2011). High prevalence of metabolic syndrome in an elderly Croatian population - a multicentre study. Public Health Nutr.

[15] Ruidavets JB, Bongard V, Dallongeville J, Arveiler D, Ducimetiere P, Perret B, Simon C, Amouyel P, Ferrieres J (2007). High consumptions of grain, fish, dairy products and combinations of these are associated with a low prevalence of metabolic syndrome. J Epidemiol Community Health.

[16] Zaribaf F, Falahi E, Barak F, Heidari M, Keshteli AH, Yazdannik A, Esmaillzadeh A (2014). Fish consumption is inversely associated with the metabolic syndrome. Eur J Clin Nutr.

[17] Alberti KG, Zimmet P, Shaw J (2006). Metabolic syndrome–a new world-wide definition. A consensus statement from the international diabetes federation. Diabet Med.

[18] Grundy SM, Cleeman JI, Daniels SR, Donato KA, Eckel RH, Franklin BA, Gordon DJ, Krauss RM, Savage PJ, Smith SC, Spertus JA, Costa F, National Heart, Lung, and Blood Institute (2005). Diagnosis and management of the metabolic syndrome: an american heart association/national heart, lung, and blood institute scientific statement. Circulation.

[19] Baik I (2009). Optimal cutoff points of waist circumference for the criteria of abdominal obesity: comparison with the criteria of the international diabetes federation. Circ J.

[20] Expert Panel on Detection E, Treatment of High Blood Cholesterol in A (2001). Executive Summary of The Third Report of The National Cholesterol Education Program (NCEP) Expert Panel on Detection, Evaluation, And Treatment of High Blood Cholesterol In Adults (Adult Treatment Panel III). JAMA.

[21] Grundy SM, Brewer HB, Cleeman JI, Smith SC, Lenfant C, American Heart A, National Heart L, Blood I (2004). Definition of metabolic syndrome: Report of the National Heart, Lung, and Blood Institute/American Heart Association conference on scientific issues related to definition. Circulation.

[22] Eckel RH, Grundy SM, Zimmet PZ (2005). The metabolic syndrome. Lancet.

[23] Akter S, Nanri A, Pham NM, Kurotani K, Mizoue T (2013). Dietary patterns and metabolic syndrome in a Japanese working population. Nutr Metab.

[24] Jung HJ, Han SN, Song S, Paik HY, Baik HW, Joung H (2011). Association between adherence to the korean food guidance system and the risk of metabolic abnormalities in koreans. Nutr Res Pract.

[25] Panagiotakos DB, Pitsavos C, Skoumas Y, Stefanadis C (2007). The association between food patterns and the metabolic syndrome using principal components analysis: The ATTICA Study. J Am Diet Assoc.

[26] Fonseca MJ, Gaio R, Lopes C, Santos AC (2012). Association between dietary patterns and metabolic syndrome in a sample of Portuguese adults. Nutr J.

[27] He Y, Li Y, Lai J, Wang D, Zhang J, Fu P, Yang X, Qi L (2012). Dietary patterns as compared with physical activity in relation to metabolic syndrome among Chinese adults. Nutr Metab Cardiovasc Dis.

[28] Alvarez Leon E, Henriquez P, Serra-Majem L (2006). Mediterranean diet and metabolic syndrome: a cross-sectional study in the Canary Islands. Public Health Nutr.

[29] Hong S, Song Y, Lee KH, Lee HS, Lee M, Jee SH, Joung H (2012). A fruit and dairy dietary pattern is associated with a reduced risk of metabolic syndrome. Metabolism.

[30] Babio N, Bullo M, Salas-Salvado J (2009). Mediterranean diet and metabolic syndrome: the evidence. Public Health Nutr.

[31] Cho YA, Kim J, Cho ER, Shin A (2011). Dietary patterns and the prevalence of metabolic syndrome in Korean women. Nutr Metab Cardiovasc Dis.

[32] Alemany M (2012). Do the interactions between glucocorticoids and sex hormones regulate the development of the metabolic syndrome?. Front Endocrinol.

[33] Cohen A, Pieper CF, Brown AJ, Bastian LA (2006). Number of children and risk of metabolic syndrome in women. J Womens Health (Larchmt).

[34] Butte NF, Wong WW, Hopkinson JM (2001). Energy requirements of lactating women derived from doubly labeled water and milk energy output. J Nutr.

[35] Butte NF, King JC (2005). Energy requirements during pregnancy and lactation. Public Health Nutr.

[36] Stuebe AM, Rich-Edwards JW (2009). The reset hypothesis: lactation and maternal metabolism. Am J Perinatol.

[37] Gunderson EP, Jacobs DR, Chiang V, Lewis CE, Feng J, Quesenberry CP, Sidney S (2010). Duration of lactation and incidence of the metabolic syndrome in women of reproductive age according to gestational diabetes mellitus status: a 20-Year prospective study in CARDIA (Coronary Artery Risk Development in Young Adults). Diabetes.

[38] Torris C, Thune I, Emaus A, Finstad SE, Bye A, Furberg AS, Barrett E, Jasienska G, Ellison P, Hjartaker A (2013). Duration of lactation, maternal metabolic profile, and body composition in the Norwegian EBBA I-study. Breastfeed Med.

[39] Vazquez C, Botella-Carretero JI, Corella D, Fiol M, Lage M, Lurbe E, Richart C, Fernandez-Real JM, Fuentes F, Ordonez A, de Cos AI, Salas-Salvadó J, Burguera B, Estruch R, Ros E, Pastor O, Casanueva FF, WISH-CARE Study Investigators (2014). White fish reduces cardiovascular risk factors in patients with metabolic syndrome: the WISH-CARE study, a multicenter randomized clinical trial. Nutr Metab Cardiovasc Dis.

[40] Uhe AM, Collier GR, O’Dea K (1992). A comparison of the effects of beef, chicken and fish protein on satiety and amino acid profiles in lean male subjects. J Nutr.

[41] El Khoury D, Anderson GH (2013). Recent advances in dietary proteins and lipid metabolism. Curr Opin Lipidol.

[42] Shukla A, Bettzieche A, Hirche F, Brandsch C, Stangl GI, Eder K (2006). Dietary fish protein alters blood lipid concentrations and hepatic genes involved in cholesterol homeostasis in the rat model. Br J Nutr.

[43] Pilon G, Ruzzin J, Rioux LE, Lavigne C, White PJ, Froyland L, Jacques H, Bryl P, Beaulieu L, Marette A (2011). Differential effects of various fish proteins in altering body weight, adiposity, inflammatory status, and insulin sensitivity in high-fat-fed rats. Metabolism.

[44] Madani Z, Louchami K, Sener A, Malaisse WJ, Ait Yahia D (2012). Dietary sardine protein lowers insulin resistance, leptin and TNF-alpha and beneficially affects adipose tissue oxidative stress in rats with fructose-induced metabolic syndrome. Int J Mol Med.

[45] Ouellet V, Marois J, Weisnagel SJ, Jacques H (2007). Dietary cod protein improves insulin sensitivity in insulin-resistant men and women: a randomized controlled trial. Diabetes Care.

[46] DiBello JR, McGarvey ST, Kraft P, Goldberg R, Campos H, Quested C, Laumoli TS, Baylin A (2009). Dietary patterns are associated with metabolic syndrome in adult Samoans. J Nutr.

[47] Baik I, Lee M, Jun NR, Lee JY, Shin C (2013). A healthy dietary pattern consisting of a variety of food choices is inversely associated with the development of metabolic syndrome. Nutr Res Pract.

[48] Di Daniele N, Petramala L, Di Renzo L, Sarlo F, Della Rocca DG, Rizzo M, Fondacaro V, Iacopino L, Pepine CJ, De Lorenzo A (2013). Body composition changes and cardiometabolic benefits of a balanced Italian Mediterranean Diet in obese patients with metabolic syndrome. Acta Diabetol.

[49] Kesse-Guyot E, Ahluwalia N, Lassale C, Hercberg S, Fezeu L, Lairon D (2013). Adherence to Mediterranean diet reduces the risk of metabolic syndrome: A 6-year prospective study. Nutr Metab Cardiovasc Dis.

[50] Lutsey PL, Steffen LM, Stevens J (2008). Dietary intake and the development of the metabolic syndrome: the Atherosclerosis Risk in Communities study. Circulation.

[51] Kim J, Jo I (2011). Grains, vegetables, and fish dietary pattern is inversely associated with the risk of metabolic syndrome in South korean adults. J Am Diet Assoc.

[52] Williams DE, Prevost AT, Whichelow MJ, Cox BD, Day NE, Wareham NJ (2000). A cross-sectional study of dietary patterns with glucose intolerance and other features of the metabolic syndrome. Br J Nutr.

[53] Amini M, Esmaillzadeh A, Shafaeizadeh S, Behrooz J, Zare M (2010). Relationship between major dietary patterns and metabolic syndrome among individuals with impaired glucose tolerance. Nutrition.

[54] Heidemann C, Scheidt-Nave C, Richter A, Mensink GB (2011). Dietary patterns are associated with cardiometabolic risk factors in a representative study population of German adults. Br J Nutr.

[55] Min C, Noh H, Kang YS, Sim HJ, Baik HW, Song WO, Yoon J, Park YH, Joung H (2011). Skipping breakfast is associated with diet quality and metabolic syndrome risk factors of adults. Nutr Res Pract.

[56] Song Y, Joung H (2012). A traditional Korean dietary pattern and metabolic syndrome abnormalities. Nutr Metab Cardiovasc Dis.

[57] Mozaffarian D, Lemaitre RN, Kuller LH, Burke GL, Tracy RP, Siscovick DS, Cardiovascular Health S (2003). Cardiac benefits of fish consumption may depend on the type of fish meal consumed: the Cardiovascular Health Study. Circulation.

[58] Patel PS, Sharp SJ, Luben RN, Khaw KT, Bingham SA, Wareham NJ, Forouhi NG (2009). Association between type of dietary fish and seafood intake and the risk of incident type 2 diabetes: the European prospective investigation of cancer (EPIC)-Norfolk cohort study. Diabetes Care.

[59] Eckel RH, Alberti KG, Grundy SM, Zimmet PZ (2010). The metabolic syndrome. Lancet.

[60] Esmailzadehha N, Ziaee A, Kazemifar AM, Ghorbani A, Oveisi S (2013). Prevalence of metabolic syndrome in Qazvin Metabolic Diseases Study (QMDS), Iran: A comparative analysis of six definitions. Endocr Regul.

